# Combinatorial Avidity Selection of Mosaic Landscape Phages Targeted at Breast Cancer Cells—An Alternative Mechanism of Directed Molecular Evolution

**DOI:** 10.3390/v11090785

**Published:** 2019-08-26

**Authors:** Valery A. Petrenko, James W. Gillespie, Hai Xu, Tiffany O’Dell, Laura M. De Plano

**Affiliations:** 1 Department of Pathobiology, College of Veterinary Medicine, Auburn University, Auburn, AL 36849, USA; 2 National Veterinary Biological Medicine Engineering Research Center, Jiangsu Academy of Agricultural Sciences, Nanjing 210014, Jiangsu, China; 3 Department of Chemical Sciences, Biological, Pharmaceutical and Environmental, University of Messina, Viale F. Stagno d’Alcontres 31, 98166 Messina, Italy

**Keywords:** landscape phage display, directed molecular evolution, combinatorial selection, short linear motifs, domain motif interaction, breast cancer

## Abstract

Low performance of actively targeted nanomedicines required revision of the traditional drug targeting paradigm and stimulated the development of novel phage-programmed, self-navigating drug delivery vehicles. In the proposed smart vehicles, targeting peptides, selected from phage libraries using traditional principles of affinity selection, are substituted for phage proteins discovered through combinatorial avidity selection. Here, we substantiate the potential of combinatorial avidity selection using landscape phage in the discovery of Short Linear Motifs (SLiMs) and their partner domains. We proved an algorithm for analysis of phage populations evolved through multistage screening of landscape phage libraries against the MDA-MB-231 breast cancer cell line. The suggested combinatorial avidity selection model proposes a multistage accumulation of Elementary Binding Units (EBU), or Core Motifs (CorMs), in landscape phage fusion peptides, serving as evolutionary initiators for formation of SLiMs. Combinatorial selection has the potential to harness directed molecular evolution to create novel smart materials with diverse novel, emergent properties.

## 1. Introduction

**Figure 1:**
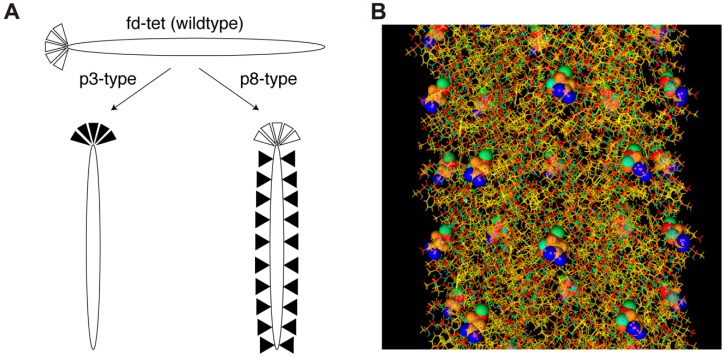
Major types of phage display libraries. (**A**) Schematic diagrams of p3-type versus p8-type phage display libraries derived from the fd-tet parent vector (display on other minor coats or hybrid type libraries are not shown). Filamentous phage, fd-tet, is represented as a thin, flexible rod ~1 µm in length by 6 nm in diameter and is composed of a circular, single stranded DNA genome encapsulated by 5 structural proteins (p3, p6, p7, p8, and p9). The most prominent protein encloses the ssDNA genome in ~4000 copies of the p8 major coat protein (depicted as an oval in the diagram). At each end of the phage, there are 5 copies of each minor coat proteins with p3 and p6 at one end and p7 and p9 at the other end (here, only the p3 protein is depicted and is shown as an open triangle). Introduction of a foreign, degenerate oligonucleotide sequence at the N-terminus of either p3 or p8 coat proteins leads to display of a peptide fusion on each respective coat protein (indicated as a filled black triangle). In type 3 libraries, the foreign fusion peptide is displayed on all 5 copies of the p3 minor coat protein. In alternative type 8 libraries—or landscape phage display libraries, the foreign fusion peptide is displayed on all 4000 copies of the p8 major coat protein leading to dramatic changes to the surface properties of each phage particle. (**B**) A stick model diagram of the landscape phage (~10 nm segment of the full length). Fusion peptide sequences displayed at the N-terminus of every copy of p8 coat protein are modeled as spheres to demonstrate the unique structural ‘landscapes’ generated on the surface of particle.

Improvements to conventional cancer chemotherapeutics are required, due largely to their random distribution throughout the patient’s body, resulting in severe adverse side-effects related to non-specific toxicity. To address the limitations of current cancer chemotherapy, it was suggested to delegate the delicate drug delivery mission to molecular smart machines, or nano-robots, that can operate without human intervention [1,2,3,4]. These nano-robots are envisioned as autonomous, mobile, nano-sized devices—able to freely move through the body to complete their mission under their own control without human intervention [5]. To operate precisely and independently, smart molecular robots should be programmed, in the same way as self-driving cars, drones and other smart robotic transportation systems [6]. To develop a molecular program for navigating smart nano-vehicles to primary and metastatic tumors, we proposed using landscape phages and their multifunctional mosaic proteins discovered by directed molecular evolution [7]. The originally postulated driving mechanism of molecular evolution, affinity selection, allows discovery of phage-displayed proteins with a high affinity towards a molecular target [8]. However, like natural evolution, molecular evolution cannot be driven only by point mutations in functional protein domains, but can be rather successful through diversification of molecular populations by modification of Short Linear Motifs (SLiMs) in disordered protein regions [9,10,11,12,13]. Thus, peptide maturation through affinity screening of diverse populations of random peptide libraries itself cannot satisfy many requirements of molecular and material bioengineers in creating new functional molecules and smart, mobile nano- and micro-sized devices. In the search for molecules able to navigate and migrate smart molecular robots in a complex multicellular environment, we propose an alternative mechanism for directed molecular evolution—combinatorial avidity selection driven by reassortments of distinct SLiMs on the surface of landscape phages [1,7,14]. SLiMs are short protein modules (3–10 amino acids long) with a 2–3 key residues directly contacting a globular domain of a protein partner [15]. These weak interactions mediate most protein–protein interactions and support a diverse set of biochemical processes, including: cell targeting, post-translational modifications, cell signaling, trafficking, protein stability, cell-cycle progression, molecular switching, protein degradation, and many other critical cellular signaling and regulation functions [12,15,16,17,18,19]. Affinity of SLiM-mediated interactions with their binding partners are normally very low (1–150 µM range) [20], which in turn allows them to participate in a dynamic network of activities, where multi-protein complexes rapidly assemble and disassemble, through reversible SLiM-mediated interactions [15]. These properties of SLiMs allowed proposing them as potential programming modules for development of self-navigating molecular robots [1]. Most molecular evolution phage display projects explore filamentous phages—long, thin viruses, consisting of a single-stranded circular DNA packed in a cylindrical shell composed of the major coat protein p8 (~90% of the virion mass and 98% of the protein mass), and a few copies of the minor coat proteins capping the ends of the phage particle (Figure 1). In phage display constructs, a foreign coding sequence is spliced in-frame into one of the phage coat protein genes, so that the foreign peptide encoded by that sequence is fused to the phage coat protein and thereby displayed on the exposed surface of the virion. A phage display library is an ensemble of up to about 10 billion such phage clones, each displaying a different guest peptide on the virion surface. Phage display libraries are commonly used to identify proteins and their fusion peptides interacting specifically and selectively with various targets, including organ tissues, tumors and their isolated cells [8]. The p3 minor coat protein and p8 major coat protein, commonly used for phage display, are presented by five copies at the phage distal end and up to 2700 copies over the virus surface (~4000 copies in fd-tet type vectors used in this project), respectively (Figure 1). P3-displayed libraries are used to select high affinity peptides and antibodies in affinity selection procedure called biopanning. In contrast, p8-expressing phages allow selection of peptides with lower affinities, as their dense arrangement on the virion’s surface results in stronger binding due to avidity masking low individual peptide affinity [21]. With our prospective goal of using phages to identify molecular programs for intercellular navigation, p8 expressing phages, or landscape phages [7], become invaluable research material because of their advantage for reversible binding to polyvalent cellular receptors through a number of short amino acid sequence motifs (hypothetical Elementary Binding Units, EBU [22] accommodated on their fusion peptides. As a first step towards identification of molecular targeting programs, which would drive the migration of phages and phage-programmed nano-devices [7] in complex multicellular systems, like tumor tissue, we studied the evolution of landscape phage libraries during their iterative multistage enrichment against breast cancer cells in vitro.

## 2. Materials and Methods

### 2.1. Cells and Cell Culture

Human cell lines used in this study were purchased from the American Type Culture Collection (ATCC, Manassas, VA, USA): MCF-7 (ATCC^®^ HTB-22™), MCF-10A (Phenotypically normal; ATCC^®^ CRL-10317™), MDA-MB-157 (Basal B/Claudin-low; ATCC^®^ HTB-24™), MDA-MB-231 (ATCC^®^ HTB-26™), MDA-MB-361 (Luminal B; ATCC^®^ HTB-27™), MDA-MB-453 (HER2^+^; ATCC^®^ HTB-131™), MDA-MB-468 (Basal A; ATCC^®^ HTB-132™). Most MDA-MB-231 cells display a basal-like phenotype (>90% CD44^+^/CD24^−^). However, a subset of the basal-like cell population is enriched for a tumor-initiating subpopulation (~2–5% CD44^+^/CD24^−^/ESA^+^) with increased capacity to generate tumors in murine models [23]. All cells were maintained as subconfluent monolayers in 25-cm^2^ polystyrene flasks in the respective complete growth medium for each cell type, as recommended by ATCC, and grown in a water-jacketed incubator at 37 °C with 5% CO_2_. A comparison of genome copy number and transcriptional profiles for the cell lines with those measured for primary breast tumors is available from [24].

### 2.2. Landscape Phage Display Library

Polyvalent, mosaic peptide phage displayed libraries, or shortly—landscape phage libraries were constructed in collaboration with Prof. George Smith (Nobel Laureate in Chemistry, 2018) [7,8,25]. In this type 8 phage display system, the guest peptide is displayed as an extension of each major coat protein due to an in-frame random oligonucleotide insertion in the gene *gpVIII* encoding the major coat protein, resulting in the display of ~4000 guest peptides on the surface of each phage particle. More specifically, in the search for self-programming phages, we used libraries f8/8 (~1.4 × 10^9^ clones) and f8/9 (~1.2 × 10^9^ clones), in which peptides EGE and EGED at the N-terminus of pVIII protein were replaced by random 8- and 9-mer peptides, as described previously [7,25,26]. All general methods of handling phage, including propagation, purification, titering, production of pure phage clone and isolation of phage DNA, were summarized previously [27].

### 2.3. Selection of Breast Cancer Cell-Specific Phages

#### 2.3.1. Depletion and First Round Selection

MDA-MB-231 breast cancer and phenotypically normal MCF-10A breast epithelial cells were cultured in 25-cm^2^ flasks until ~90% confluent. An aliquot of each library containing ~10^11^ virions, with each unique fusion sequence being represented by ~100 copies, was diluted in blocking buffer (DMEM supplemented with 10% FBS + 0.5% BSA) and transferred to an empty 25-cm^2^ cell culture-treated flask for one hour at room temperature to deplete the library of phages adsorbing to the plastic flask. Unbound phages were recovered and transferred to a flask, treated overnight with complete growth medium (DMEM supplemented with 10% FBS) for one hour at room temperature to deplete medium- and serum-binding phages. Again, unbound phages were recovered and incubated in a confluent flask of normal breast epithelial cells, MCF-10A, for one hour at room temperature. Depleted libraries were then transferred to flasks containing confluent target MDA-MB-231 breast cancer cells and allowed to incubate for one hour at room temperature. Cells were washed and phage recovered, as in Section 2.3.2.

#### 2.3.2. Washing and Sublibrary Generation

Unbound phages were recovered from each flask and saved for titering. MDA-MB-231 cell monolayers were washed for five minutes with cold (4 °C) washing buffer (DMEM with 0.1% Tween 20/0.5% BSA) for a total of ten washes to remove low binding phage. Washes were collected and saved for titering. Surface bound “eluate” phages were recovered by incubation in elution buffer (200 mM glycine, pH 2.2/0.1% BSA) for 10 min followed by neutralization with neutralizing buffer (1.0 M Tris-HCl, pH 9.1). Adherent cells were washed for 5 min twice with washing buffer at room temperature and collected as “post-elution wash” fractions for titering. Remaining adherent cells were scrapped from the flask and transferred to a centrifuge tube. Cells were pelleted, the supernatant discarded, and the remaining cell pellets lysed with deoxycholate lysis buffer (2% *w*/*v* sodium deoxycholate/10 mM Tris-HCl, pH 8.0/2.0 mM EDTA) for 10 min at room temperature to isolate internalized or membrane-associated phages. Recovered eluate and post-elution wash fractions were concentrated to ~0.2 mL using Amicon 100 kDa MWCO concentrators (EMD Millipore, Billerica, MA, USA). Concentrated phages from eluate and post elution wash fractions were combined into a single eluate sublibrary fraction for each library and round of selection. Phage populations from eluate and lysate sublibrary fractions were infected into K91BluKan *E. coli* cells, amplified, and purified by PEG/NaCl precipitation for future rounds of selection. All recovered fractions were titered and quantified as described previously [27].

#### 2.3.3. Second, Third and Fourth Rounds

For additional rounds of selection, an aliquot of ~10^11^ virions from each of the eluate and lysate sublibrary fractions generated from the previous round were diluted in DMEM with 10% FBS and incubated with confluent MDA-MB-231 cells in a 37 °C cell culture incubator with 5% CO_2_ for one hour. Cells were washed and phage recovered as in Section 2.3.2.

#### 2.3.4. Sequencing of Sublibraries

Following the final round of selection (3rd for f8/8 library and 4th for f8/9 library), a portion of the phage-infected bacterial culture was spread on an NZY/Kan/Tet agar plate after 45 min of growth and placed in an 37 °C incubator overnight. *E. coli* colonies containing individual phage clones were randomly picked, gridded onto NZY/Kan/Tet agar plates and incubated at 37 °C overnight. The DNA segment containing the sequence of *gpVIII* was amplified by PCR as described [27]. PCR products were purified and sequenced by dye-terminator sequencing at the Massachusetts General Hospital (MGH) DNA Core (Cambridge, MA, USA). Unique clones were propagated in 2 mL scale and purified/concentrated by double PEG/NaCl precipitation for archiving and future experiments.

### 2.4. Computational Analysis

The *gp8* DNA sequences from unique phage clones were translated to their corresponding p8 fusion protein sequences using EditSeq tool of the DNASTAR, ver.11 (Madison, WI, USA) suite of molecular biology analysis programs. The resulting list of fusion peptides displayed on the phage proteins (peptide inserts) was converted into FASTA files using Excel 2013, and was analyzed with MEME Suite (Motif-based sequence analysis tools v.5.0.5 [28]; http://meme-suite.org/) to discover non-degenerate, non-gapped, tri-peptide motifs. For convenience, phages were designated by the sequence of the displayed guest p8-fusion peptide. For example, phage isolated from the f8/8 phage landscape library, harboring ~4000 copies of 55-mer fusion coat protein ADMPGTVLPDPAKAAFDSLQASATEYIGYAWAMVVVIVGATIGIKLFKKFTSKAS was designated as DMPGTVLP. Similarly, phage isolated from f8/9 library and harboring 55-mer fusion protein ADRDDSFMNDPAKAAFDSLQASATEYIGYAWAMVVVIVGATIGIKLFKKFTSKAS was designated as DRDDSFMND. The likely range of SLiMs, and Domain Motif Interactions (DMIs) they mediate, were identified using the Eukaryotic Linear Motif (ELM) resource [12], http://elm.eu.org/. Biopanning Data Bank BDB [29], http://immunet.cn/bdb/index.php, a manually curated, publicly accessible database of peptides, selected from random phage display libraries, was used for identification of conserved structural motifs within short peptides, and for their comparison to the latest released version of the database. For prediction of protein functional activity, classifying peptides into families and predicting domains and important sites, we used InterPro—a single searchable resource that combines protein signatures from multiple databases [30], http://www.ebi.ac.uk/interpro/. For annotation of functional attributes that can be assigned to a genome, based on the presence of a defined set of protein family markers within that genome, we used an annotation system “Genome properties (GP)” [31], https://www.ebi.ac.uk/interpro/genomeproperties/.

### 2.5. Specificity and Selectivity of Phages

Individual representative phage clones, identified by DNA sequencing, were propagated and purified, as described [27], to be used in cell-association assays [32,33]. In a primary semi-quantitative screening assay, we tested the association of each selected clone with the target MDA-MB-231 breast cancer cells in comparison with serum. Briefly, phage particles (~10^6^ CFU/well) were incubated with target cells- and serum-treated control wells in a 96-well cell culture plate. Following several washes, cell- or serum-associated phages were collected by treating each well with CHAPS lysis buffer and titering in K91BluKan *E. coli* cells. Binding efficiency of individual phages was calculated as the percent ratio of output CFU to input phage. The most prospective phage binders, i.e., those phages that demonstrated increased binding to target MDA-MB-231 cells rather than serum components, were further tested for their ability to discriminate between different targets (selectivity) using a panel of various breast cancer cell subtypes. Shortly, target MDA-MB-231 breast cancer, MCF-10A normal breast epithelial cells, and control breast cancer cells MCF-7, MDA-MB-157, MDA-MB-361, MDA-MB-453, and MDA-MB-468 were grown to confluence in separate wells of a 96-well cell culture-treated plate. As a control, some wells were treated with media alone. Before application of phages, media in each well was replaced with serum-free media pre-warmed at 37 °C and incubated for one hour in a 37 °C incubator with 5% CO_2_. Each phage clones (~10^6^ CFU/well) was deposited in the designated wells in 100 μL of pre-warmed blocking buffer and incubated for one hour at 37 °C. Unbound phages were carefully removed and wells were washed eight times with 100 μL of washing buffer pre-warmed to 37 °C. To collect cell-associated phages, 25 μL of CHAPS lysis buffer (2.5% *w*/*v* CHAPS [3-((3-cholamidopropyl)dimethylammonio)-1-propanesulfonate] in DMEM/F12) was added to each well and incubated for 10 min on a shaker with gentle rocking. A portion 125 μL of starved K91BluKan *E. coli* were added to each well and incubated for 15 min at room temperature. Next, 180 μL of NZY/Tet (0.4 μg/mL) was added to the mixture and incubated for 45 min in a 37 °C incubator. The final mixture was spread on NZY/Kan/Tet agar plates and incubated overnight in a 37 °C incubator. Phage recovery was calculated as the percent ratio of output TU to input TU. A previously identified nucleolin-binding phage displaying the fusion peptide DMPGDVLP was used as a positive control that demonstrates specific binding activity towards MCF-7 cells [33]. A non-related streptavidin-binding phage bearing an unrelated peptide VPEGAFSS [34] was used as a negative control. All selectivity and specificity cell-associated assays were performed in triplicate with data reported as the mean ± standard deviation.

### 2.6. Immunofluorescence Analysis of Phages

Interactions of isolated phage clones with MDA-MB-231 breast cancer cells were analyzed as previously [35]. Briefly, MDA-MB-231 cells were seeded into 4-well chamber slides (~50,000 cells/well in L15 medium) and incubated in a 37 °C incubator with 5% CO_2_ until cells were ~70% confluent. Cells were washed 3 times with 1× PBS, pH 7.4 for 5 min at room temperature. Next, cells were incubated with ~1.0 × 10^10^ virions of an isolated phage clone in serum-free L15 culture medium for 15 min or up to 24 h at 37 °C. Cells were washed with 1× PBS, pH 7.4 and fixed with 4% paraformaldehyde in PBS for 15 min at room temperature. After an additional 3 washes, cells were permeabilized with 0.1% Triton X-100 in PBS for 10 min at room temperature and blocked with 1% BSA for 30 min at room temperature. Cells were treated with a 1:1000 dilution of 3.3 mg/mL rabbit anti-fd bacteriophage antibodies [36] in blocking buffer (1% BSA in 1× PBS, pH 7.4) for 1 h at room temperature. Cells were washed with 1× PBS and treated with a 1:500 dilution of AlexaFlour^®^ 488 goat anti-rabbit IgG and 0.022 µM AlexaFlour^®^ 546 phalloidin for 1 h at room temperature in the dark. After washing, slides were cover slipped with VECTAshield mounting medium with DAPI (Vector Laboratories). Slides were visualized with a Nikon A1 laser module coupled to a Nikon Eclipse C1 2000-E confocal microscope and z-stacks captured using the Nikon Elements software package at 0.150 µm/step with representative slices shown.

## 3. Results

### 3.1. Selection of Breast Cancer Cell-Specific Landscape Phages

For discovery of phage proteins, self-programmed to bind and penetrate into cancer cells, we used multibillion-clone landscape phage libraries f8/8 and f8/9, whose performance as an enormous reservoir of phage particles interacting with human cancer cells was demonstrated previously [7]. As a target for selection, we used the human metastatic breast cancer cell line MDA-MB-231, originally established from a pleural effusion from a patient presenting with a metastatic mammary adenocarcinoma [37]. These cells are an ideal model for late stage, triple negative breast cancer (ER^−^, PR^−^, and HER2^−^) with a highly aggressive phenotype and poor clinical prognosis. To isolate phage peptides with high selectivity towards the MDA-MB-231 target cells, we adopted a rigorous library depletion protocol in which the naive library was progressively depleted against plastic, serum and phenotypically normal breast epithelial cells before interacting with the target cells [14,32,33,38]. Following the final round of selection, hundreds of phage clones from the eluate, lysate, and post-elution wash fractions were randomly chosen for PCR amplification with phage-specific primers and determination of the *gp8* nucleotide sequence by Sanger sequencing. FASTA files containing structurally unique peptide sequences were analyzed using the Multiple EM Motif Elicitation MEME program, a component of the MEME Suite collection of tools, to reveal linear, non-gapped, tri-mer motifs which we identified as Core Motifs or CorMs (Table S1).

### 3.2. Specificity and Selectivity of Phages Towards Breast Cancer Cells

**Figure 2:**
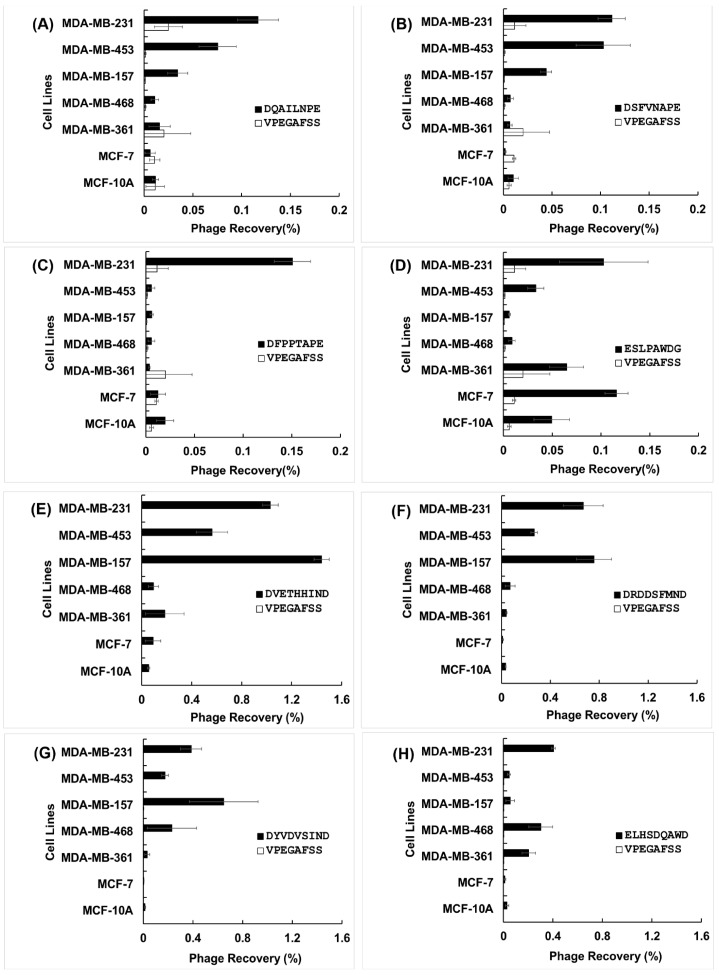
Selectivity of phages towards cancer cell lines. Binding of selected phages towards the target cell line, MDA-MB-231, in comparison with other cell lines derived from different breast cancer subtypes (MCF-7, MDA-MB-453, MDA-MB-157, MDA-MB-468, MDA-MB-361) and the phenotypically normal breast epithelial cell line (MCF-10A). (**A**–**D**) Phage clones selected from the f8/9 phage library and (**E**–**H**) phage clones selected from the f8/9 phage library. An unrelated phage bearing the peptide sequence VPEGAFSS was used as a negative control for each assay. Specificity of the phages were estimated as their percent recovery (%) = output phage/input phage × 100.

The high throughput cell-association assay [32] was used to evaluate both specificity of selected phages (in comparison with non-related phage), and their selectivity towards target cancer cells in comparison with other breast cancer cell lines (Figure 2). In characterizing phage clones, we define specificity as the ability of a phage probe to associate with its target due to the presence of a specific peptide sequence displayed on the surface of the phage, whereas selectivity is the ability of a phage probe to discriminate its cognate target from a mixture of targets.

**Figure 3:**
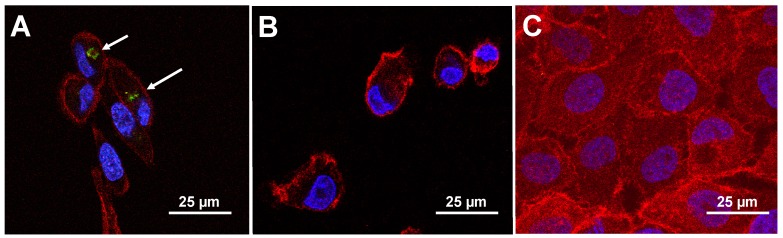
Immunofluorescence analysis of phages towards breast cancer cells in vitro. Phage particles (~10^10^ virions) displaying (**A**) a breast cancer cell-specific peptide DSFVNAPE or (**B**) an unrelated peptide VPEGAFSS were incubated with MDA-MB-231 breast cancer cells for 1 h and visualized at 60× magnification using confocal microscopy. (**C**) Similarly, phage displaying the breast cancer cell-specific peptide DSFVNAPE as incubated with MCF-10A cells for 1 h. Phages were revealed using a rabbit anti-fd IgG primary antibody and an AlexaFluor^®^ 488-conjugated goat anti-rabbit IgG secondary antibody (green), actin was identified using AlexaFluor^®^ 546-labeled phalloidin (red) and nuclei were identified by DAPI staining (blue). (**A**) DSFVNAPE phages (identified by white arrows) were found internalized after 1 h and demonstrated perinuclear localization as round spots. Linear phage particles could also be found on the surface of the cells in other time points. (**B**) Phages bearing an unrelated peptide were not found interacting with the cells under the same conditions. (**C**) DSFVNAPE phages were not found interacting with the phenotypically normal breast epithelial cell line, MFC-10A, under the same conditions.

We analyzed the most specific and selective phage clones further using in silico methods to reveal structural origins of their behavior, as summarized in Supplemental Table S1. To find the origins of specificity and selectivity of selected phages towards different cancer cells, we analyzed a panel of distinct CorMs (revealed as SLiMs) in interaction of landscape phages with corresponding protein domains on/in cancer cells. For example, the phage clone displaying the DSFVNAPE peptide sequence exhibits the greatest selectivity toward MDA-MB-231 and MDA-MB-453 (~10-fold) in comparison with phenotypically normal breast epithelial cells, MCF-10A (Figure 2). The specific structural features of this clone are revealed by the combination of five CorMs (DSF, FVN, VNA, APE, PED) in the displayed fusion peptide, which were enriched through combinatorial selection into two functional SLiMs (DSFVNAP and VNAPEDP), responsible for interaction of the fusion peptide with a SRC Homology 3 (SH3) domain with non-canonical class I specificity (XXX[PV]XXP) [39,40] (Supplemental Table S1). Classically, SH3 domains are present in all eukaryotes and are restricted to intracellular proteins involved in regulation of cellular signaling pathways, substrate recognition, and membrane localization [41]. However, the small human melanoma inhibitory activity (MIA) protein was the first extracellular protein discovered containing an SH3 domain-like fold [42] and suggesting that additional extracellular proteins with SH3 domain-like folds may exist. Thus, we suppose that phage DSFVNAPE may be found either extracellularly and/or intracellularly. The intracellular localization of the phage was evidenced by confocal florescent microscopy (Figure 3). Specificity of this imaging method was confirmed in a negative control test with nonrelated streptavidin-binding phage VPEGAFSS (Petrenko and Smith, 2000).

Another phage DFPPTAPE demonstrating strong selectivity towards MDA-MB-231 cells (~8-fold stronger binding over MCF-10A cells) contains five CorMs (DFP, FPP, PPT, APE, PED) within the fusion peptide and demonstrates enrichment of three SLiMs (PPTAPE, PEDPAK and FPPTAPE) that can interact via a PTAP binding site within the UEV domain (XP[TS]APX), a SH3-domain with class II specificity (PXXPX[KR]) and a CK2 phosphorylation site (XXX[ST]XXE), respectively (Supplemental Table S1). The PTAP motif is responsible for mediating weak binding of several cellular proteins to the ubiquitin E2 variant domain (UEV) of the tumor susceptibility gene 101 (Tsg101) protein. Under normal physiologic conditions, Tsg101 is a required component of the Endosomal Sorting Complexes Required for Transport I (ESCRT-I) complex, binding to ubiquitinated cargo proteins and sorting cargos into multivesicular bodies for cellular transport. Tsg101 recognizes ubiquitinated cargoes via an ~145 amino acid N-terminal UEV domain [43] and then recruits the remaining downstream proteins in the ESCRT-I complex. The PTAP binding site functions independently from the ubiquitin binding site in the UEV domain [44] and has been demonstrated to play an important role in virus budding [45] for several enveloped RNA viruses including HIV [46], Vesicular Stomatitis Virus [47], and Ebola virus [48]. We can hypothesize that a mechanism of ‘phage budding’ can drive intracellular sorting of phage encapsulated endosomes to transcytosis pathways, facilitating delivery of phage-programed vehicles across cellular barriers [49].

Four phage clones isolated from f8/9 landscape phage library: ELHSDQAWD, DRDDSFMND, DVETHHIND and DYVDVSIND, exhibit 14-fold, 20-fold, 26-fold, and 18-fold selectivity for MDA-MB-231 cells over MCF-10A cells, respectively. The origins of their strong binding and selectivity could not be readily explained. We identified one potential CorM (DQA) from phage ELHSDQAWD and no enrichment for any SLiMs; four CorMs (DRD, RDD, DDS, DSF) and one SLiM (RDDSFMN) were enriched in phage DRDDSFMND; two CorMs (DVE, IND) and one SLiM (VETHHIN) were enriched in phage DVETHHIND; and two CorMs (VDV, IND) and one SLiM (DYVDV) were enriched in phage DYVDVSIND. Potential target binding domains for these SLiMs are summarized in Table 1. The SLiM RDDSFMN identified in phage DRDDSFMND is proposed to interact with a polo-box domain (PBD) found within the non-catalytic C-terminus of Polo-like kinase 1 (Plk-1) and Polo-like kinase 4 (Plk-4). The PBD serves as an essential mediator of protein-protein interactions bringing the kinase domain of Plk1 into close proximity with its substrates, mainly through phosphorylation activity [50]. Plks are a class of critical kinases involved in eukaryotic cell division including regulation of the G2/M transition, mitotic entry/exit, spindle assembly, centrosome maturation, chromosome segregation, cell cycle arrest, and cytokinesis [51]. Plks are characterized as having a conserved N-terminal kinase domain (KD) linked to a C-terminal domain with one or more polo box domains (PBDs) which mediates protein interactions with targets and regulates the activity of the kinase domain. Aberrant expression of Plk-1 is strongly associated with development of many types of cancer including breast cancers and is related to a poor clinical prognosis [52]. Selective inhibition of Plk-1 has been suggested as a potential therapeutic target for development of future chemotherapies [51]. The potential target for phage DVETHHIND is the forkhead-associated domain (FHA domain) found in many regulatory proteins [53]. FHA domain normally recognizes phosphothreonine containing peptides on the ligand proteins and is prevalent in nuclear proteins that are involved in cell cycle checkpoint, DNA repair and transcriptional regulation. Thus, to be involved in interaction with FHA domain, phage proteins should be first phosphorylated with kinases. The potential target for the phage DYVDVSIND is a spectrum of protein domains: GRB2-like Src Homology 2 (SH2) domain; the autophagy-related protein Atg8; the WD40 repeat domain of WDR5 protein; Polo-like kinase PLK-1; and Tyrosine-based sorting signal responsible for the interaction with mu subunit of AP (Adaptor Protein) complex. All these interactions of phage with intracellular targets can be controlled by SLiMs DYVD and YVDV.

Phages DDTIALLNE and EELEHLLNE contain the same CorMs LLN and LNE, but different SLiMs (DTIALL, TIALLNE, DTIAL and ELEHLLN, ELEHLL correspondingly). SLiM TIALLNE specifies a possible association of phage DDTIALLNE with SUMO Type I transmembrane proteins, responsible for sorting and internalization signal and locating at the cytoplasmic juxta-membrane region. SLiM DTIALL can specify also its interaction with sorting and internalization signal found in the cytoplasmic juxta-membrane region of type I transmembrane proteins and adaptor protein (AP) complexes. Its phosphothreonine motif can bind a subset of FHA domains. Meantime, SLiM ELEHLL point to the likely binding of phage EELEHLLNE to the cytoplasmic juxta-membrane region of type I transmembrane proteins and adaptor protein (AP) complexes. This diversity of possible targets for these phages can explain different spectrum of their selectivity: while phage DDTIALLNE exhibits high selectivity for MDA-MB-231, MDA-MB-361 and MDA-MB-453 belonging to different subtypes (18-, 18-, and 27-fold respectively), phage EELEHLLNE is equally selective to cells MDA-MB-231 and MDA-MB-157, both belonging to Claudin-low subtype [54].

Phages VHPDDSYSD (SLiM PDDSY; CorMs VHP, DDS, DSY, SYS and YSD), and GTGPLDSYD (SLiM PLDSY; CorMs GTG, DSY) contain the same motif [P or A][none P][none FYWIL][S][none P], which is present in the previously discovered phage EPTHSWAT [35]. This phage was shown to migrate from the culture through the membrane of prostate cells PC-3M, accumulate in cytoplasm and ultimately move to the nuclei of the cells. The migration of the phage can be explained by the presence of the motif PTHSW that can bind MATH domain, which is responsible for substrate recognition and nuclear localization of USP7—an enzyme that cleaves ubiquitin moieties from its substrates. Examples of domain-SLiM complexes, discovered during multistage evolution of landscape libraries, are shown in Table 1. They include special domains, such as PDZ, SH2, SH3, WW, etc., which are known to assemble constituent proteins into large complexes, bringing together different combinations of catalytic domains with regulatory domains [55].

## 4. Discussion

**Figure 4:**
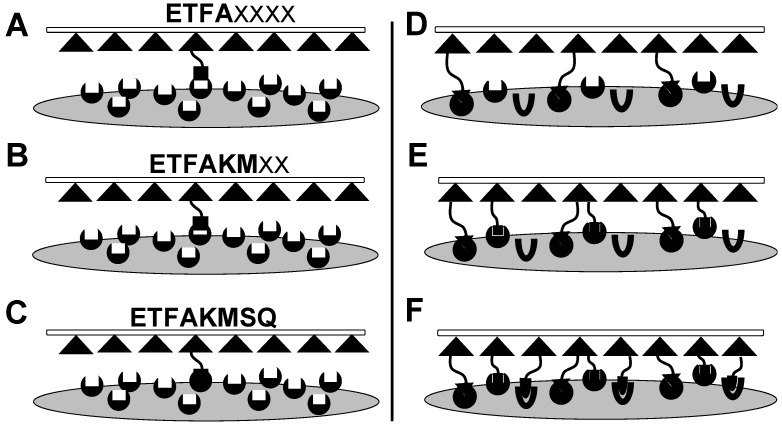
Affinity maturation (**A**–**C**). Selection of phage peptide sequences based on monovalent interactions with an immobilized target. Populations are enriched by increasing the stringency of a selective pressure, in this example binding to an immobilized target, through multiple rounds of selection. (**A**) In the first round of selection, phage bind through relatively weak, monovalent interactions as populations of phage compete for a primary binding site involving relatively few residues. (**B**,**C**) In the second and subsequent rounds of selection, the stringency is increased for improved binding, which results in phage populations with involvement of more residues in the ligand-target binding site and increased affinity towards the target. Combinatorial avidity selection (**D**–**F**). Enrichment of SLiMs based on multiple, low affinity, multivalent interactions between neighboring targets within the same particle. Populations are enriched by accumulation of multiple core motifs (CorMs) with low affinity, but increasing overall avidity, as the result of multiple, multivalent ligand-target interactions in the same ligand. (**D**) CorMs enriched in the first round of selection may either proceed through successive rounds by the traditional affinity maturation to form active SLiMs. (**E**) Alternatively, active SLiMs may be enriched by shuffling CorMs that increase avidity to the complex target surface. Here, two theoretical CorMs (triangle and square) are displayed on particles with the first interacting CorM. When no additional targets are free to bind available ligands, avidity should increase through binding of a second CorM located on the same particle that increases fitness through successive rounds of selection with stronger stringency. (**F**) Additional CorMs may also accumulate (oval) to increase the total avidity of the particle.

Affinity selection—the traditional selection paradigm [8,56] starts with a peptide or antibody phage-displayed library and seeks to discover the phages that bind strongly and specifically to a specified target immobilized on a solid surface. Unbound phage particles are removed and bound virions are eluted in an infective form, yielding an “eluate” fraction—an enriched subset of the original phage library for binding to the immobilized target. The eluate fraction, after propagation in host bacteria to generate an eluate sublibrary, is used as input in the subsequent rounds of selection. To illustrate the principles of affinity selection (Figure 4A–C), we present data (Table 1) obtained previously following multistage affinity selection of landscape phage experiments using an individual immobilized antigen, ß-Galactosidase [34]. Evolution of binding phage populations in this example is driven by the stringency of selection conditions, that results in the discovery of lead peptides DTFAKSMQ, DTFAKMSQ and DTFAKMTQ, which demonstrate strict structural homology and highest affinity to the target receptor—β-galactosidase. A collection of structural homologues to the lead peptides forms a family of motifs (medium and weak binders depicted as bold on grey background, Table 1) with decreased affinity to the target confirmed by ELISA [34].

**Figure 5:**
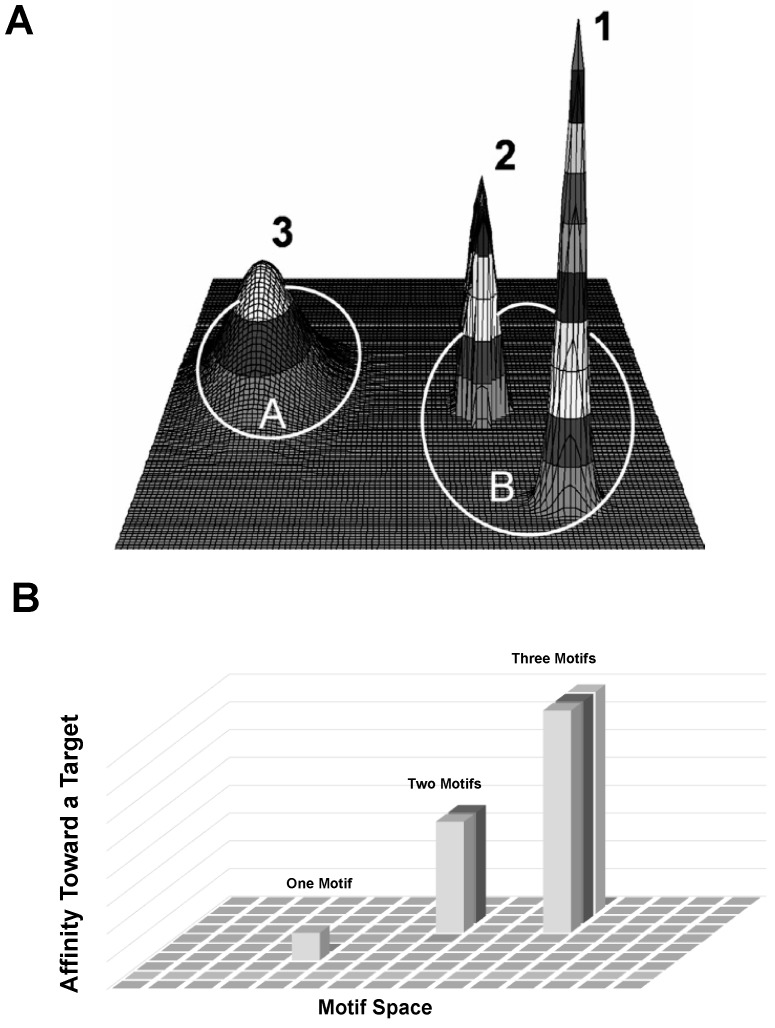
Three-dimensional representation of peptide populations in a theoretical sequence space. (**A**) Adapted from [26]. A highly simplified two-dimensional table of all possible combinations of amino acids for a given number of randomized positions can be represented as a grid with each position in the grid standing for a unique combination of amino acids. Phage libraries would be seen as a collection of random points in the sequence space and be expected to form overlapping domains within such a grid as depicted in labeled circles A and B. An additional dimension is superimposed onto the grid when libraries are considered in their context of affinity towards a target. When a structurally related peptide clusters on the basis of their target affinity, an ‘affinity cone’ is generated (peaks 1, 2, and 3) with affinity towards the target increasing from the base of the cone to the peak of the cone until a ‘lead peptide’ with highest affinity is identified. (**B**) Following a previously suggested model [8], a highly simplified two-dimensional table of all possible combinations of EBUs for a given number of randomized positions can be represented as a grid with each position of a grid standing for a unique combination of EBUs. For simplicity, we assume that points that are close together represent similar functioning EBUs. Here, we illustrate the possible sequences for a hypothetical library with two non-overlapping sets of randomized EBUs along the axes of the planes for ~60,000 (20 × 20 × 20 × 7) of possible tripeptides. An additional dimension is superimposed onto the grid when phage libraries are considered for their affinity towards a given complex target. As the number of EBUs combine to form functional SLiMs their affinity is enriched as demonstrated by the accumulation of taller peaks with each added EBU into the total peptide structure.

In a graphic model of such relationships accrued during affinity selection [8], the ensemble of all possible combinations of amino acids at randomized positions in a library comprises of an abstract geometric domain that is commonly called a sequence space (Figure 5). In this sequence affinity cone, any individual sequence is a point, with the lead peptide sequence occupying the peak and members of the family occupying descending levels in the cone based on a hierarchical gradient of affinity to the target.

Meantime, many important cellular processes involve transient, low- to moderate-affinity protein-protein interactions (PPIs) mediated by SLiMs in one protein interacting with a globular domain in another [55]. Despite their significance in many cellular processes, these domain-motif interactions (DMIs) are typically low affinity, making them challenging to identify by classical experimental approaches, including traditional phage display affinity selection and affinity maturation techniques. As a result, DMIs are generally underrepresented in PPI networks [12]. Previously, we discovered the cancer cell-binding peptides consisting of several distinct short linear motifs, which we called Elementary Binding Units (EBU), following [9,22]. EBUs were discovered during combinatorial avidity selection using an in vitro model of multicellular migration [14,57] during iterative rounds of landscape phage library screening (Figure 4D–F). We hypothesized that these short liner motifs were likely to be collected in the displayed peptides in the processes of phage-involved combinatorial molecular evolution during inter- and intracellular transportation of the landscape phage libraries. Translated to the targeted drug delivery problem, this novel approach promises to replace the existing point-to-point targeting concept for the novel phage-programmed, self-navigating drug delivery paradigm [57]. Here, this concept was further justified by analysis of a population of landscape phages that evolved from landscape libraries f8/8 and f8/9 [25,26] through their multistage screening against breast cancer cells MD-MBA-231. We discovered that EBUs revealed using purely statistical programs, such as MEME Suite [28] may well serve as Core Motifs (CorMs) in creating functional SLiMs, which we identified, along with their protein domain partners, using Eukaryotic Linear Motif (ELM) resource [12].

We suggested the discovered CorMs, SLiMs and their specific combinations as unique molecular programs, which can drive migration of landscape phages and phage-programmed nano-robots in tumor tissue, their penetration into cancer cells and finally—delivery of their cargo to the point of action. The proposed alternative mechanism of molecular evolution is based on the observation that protein-protein interactions are often mediated through SLiMs, which are defined by a consensus pattern that captures the key residues, CorMs, involved in binding to partner protein domains. In contrast to the conventional maturation affinity selection model that includes an astronomic number of possible random peptides in the sequence space, the suggested alternative combinatorial evolution selection model operates with the sequence space of landscape libraries, which can include 8000 (20 × 20 × 20) of possible tripeptides (CorMs) precursors of autonomous protein SLiMs, accommodated in different positions (up to 7) of the p8 fusion peptide. We proposed that multifunctional landscape phage particles discovered through a proposed combinatorial mechanism of molecular evolution [7], would be potentially able to migrate through the molecular/cellular barriers surrounding tumors, penetrate into the tumor mass and attack the diverse tumor cell population [1]. In this work, we demonstrate a significant potential of landscape phage technology in the discovery of SLiMs and their counter-domains using a novel algorithm of analysis of selected phage population. The suggested model proposes a combinatorial accumulation of CorMs in landscape phage fusion peptides. It was shown that CorMs serve as previously postulated Elementary Binding Units (EBU) for initiation of evolutionary formation of SLiMs. The novel combinatorial selection mechanism dramatically increases the potential for directed evolution [58] to create smart materials with novel, emergent properties. The algorithm described here for analysis of landscape phages population evolved during combinatorial selection in multicellular system, provides a solid theoretical basis for modelling and engineering molecular probes that will be used for studying and controlling various biological systems, including cellular and organ pathologies and tumor microenvironment.

## Figures and Tables

**Table 1:** Families of β-Galactosidase-binding phages grouped by relative affinity.

Strong	Medium	Weak
**DTFAK** **S** **MQ**	DTFAKSAS	ETFAASRR
**ETFAKM** **S** **Q**	DTFAKSNA	ETFASRSS
**ETFAKM** **T** **Q**	ETFARSQS	ETFAASNR
DTFAKMAQ	DTFARQNA	ETFASTRS
	ETFAKSNA	
	DTFARTQS	
	ETFARSNS	
	DTFARSSS	
	ETFASRSQ	

## Supplementary Materials

Supplementary materials can be found at https://www.mdpi.com/1999-4915/11/9/785/s1. Table S1: Target domains identified from selected phages.

## References

[1] Petrenko V.A. (2017). Autonomous self-navigating drug-delivery vehicles: From science fiction to reality. Ther. Deliv..

[2] Gao W.W., de Avila B.E.F., Zhang L.F., Wang J. (2018). Targeting and isolation of cancer cells using micro/nanomotors. Adv. Drug Deliv. Rev..

[3] Srivastava S.K., Clergeaud G., Andresen T.L., Boisen A. (2019). Micromotors for drug delivery in vivo: The road ahead. Adv. Drug Deliv. Rev..

[4] Chen H.B., Gu Z.J., An H.W., Chen C.Y., Chen J., Cui R., Chen S.Q., Chen W.H., Chen X.S., Chen X.Y. (2018). Precise nanomedicine for intelligent therapy of cancer. Sci. China Chem..

[5] Schuerle S., Soleimany A.P., Yeh T., Anand G.M., Haberli M., Fleming H.E., Mirkhani N., Qiu F., Hauert S., Wang X. (2019). Synthetic and living micropropellers for convection-enhanced nanoparticle transport. Sci. Adv..

[6] Abendroth J.M., Bushuyev O.S., Weiss P.S., Barrett C.J. (2015). Controlling motion at the nanoscale: Rise of the molecular machines. ACS Nano.

[7] Petrenko V.A. (2018). Landscape phage: Evolution from phage display to nanobiotechnology. Viruses.

[8] Smith G.P., Petrenko V.A. (1997). Phage display. Chem. Rev..

[9] Neduva V., Russell R.B. (2005). Linear motifs: Evolutionary interaction switches. FEBS Lett..

[10] Zhao H., Giver L., Shao Z., Affholter J.A., Arnold F.H. (1998). Molecular evolution by staggered extension process (step) in vitro recombination. Nat. Biotechnol..

[11] Davey N.E., Cyert M.S., Moses A.M. (2015). Short linear motifs—Ex nihilo evolution of protein regulation. Cell Commun. Signal..

[12] Gouw M., Michael S., Samano-Sanchez H., Kumar M., Zeke A., Lang B., Bely B., Chemes L.B., Davey N.E., Deng Z. (2018). The eukaryotic linear motif resource—2018 update. Nucleic Acids Res..

[13] Li X.H., Babu M.M. (2018). Human diseases from gain-of-function mutations in disordered protein regions. Cell.

[14] Gross A.L., Gillespie J.W., Petrenko V.A. (2016). Promiscuous tumor targeting phage proteins. Protein Eng. Des. Sel..

[15] Davey N.E., Van Roey K., Weatheritt R.J., Toedt G., Uyar B., Altenberg B., Budd A., Diella F., Dinkel H., Gibson T.J. (2012). Attributes of short linear motifs. Mol. Biosyst..

[16] Diella F., Haslam N., Chica C., Budd A., Michael S., Brown N.P., Trave G., Gibson T.J. (2008). Understanding eukaryotic linear motifs and their role in cell signaling and regulation. Front. Biosci. Landmrk..

[17] Van Roey K., Gibson T.J., Davey N.E. (2012). Motif switches: Decision-making in cell regulation. Curr. Opin. Struct. Biol..

[18] Van Roey K., Dinkel H., Weatheritt R.J., Gibson T.J., Davey N.E. (2013). The switches. Elm resource: A compendium of conditional regulatory interaction interfaces. Sci. Signal..

[19] Van Roey K., Uyar B., Weatheritt R.J., Dinkel H., Seiler M., Budd A., Gibson T.J., Davey N.E. (2014). Short linear motifs: Ubiquitous and functionally diverse protein interaction modules directing cell regulation. Chem. Rev..

[20] Davey N.E., Seo M.H., Yadav V.K., Jeon J., Nim S., Krystkowiak I., Blikstad C., Dong D., Markova N., Kim P.M. (2017). Discovery of short linear motif-mediated interactions through phage display of intrinsically disordered regions of the human proteome. FEBS J..

[21] Knez K., Noppe W., Geukens N., Janssen K.P., Spasic D., Heyligen J., Vriens K., Thevissen K., Cammue B.P., Petrenko V. (2013). Affinity comparison of p3 and p8 peptide displaying bacteriophages using surface plasmon resonance. Anal. Chem..

[22] Meszaros B., Simon I., Dosztanyi Z. (2009). Prediction of protein binding regions in disordered proteins. PLoS Comput. Biol..

[23] Fillmore C.M., Kuperwasser C. (2008). Human breast cancer cell lines contain stem-like cells that self-renew, give rise to phenotypically diverse progeny and survive chemotherapy. Breast Cancer Res..

[24] Neve R.M., Chin K., Fridlyand J., Yeh J., Baehner F.L., Fevr T., Clark L., Bayani N., Coppe J.P., Tong F. (2006). A collection of breast cancer cell lines for the study of functionally distinct cancer subtypes. Cancer Cell.

[25] Petrenko V.A., Smith G.P., Gong X., Quinn T. (1996). A library of organic landscapes on filamentous phage. Protein Eng..

[26] Kuzmicheva G.A., Jayanna P.K., Sorokulova I.B., Petrenko V.A. (2009). Diversity and censoring of landscape phage libraries. Protein Eng. Des. Sel..

[27] Brigati J.R., Samoylova T.I., Jayanna P.K., Petrenko V.A. (2008). Phage display for generating peptide reagents. Curr. Protoc. Protein Sci..

[28] Bailey T.L., Boden M., Buske F.A., Frith M., Grant C.E., Clementi L., Ren J., Li W.W., Noble W.S. (2009). Meme suite: Tools for motif discovery and searching. Nucleic Acids Res..

[29] He B., Chai G., Duan Y., Yan Z., Qiu L., Zhang H., Liu Z., He Q., Han K., Ru B. (2016). Bdb: Biopanning data bank. Nucleic Acids Res..

[30] Mitchell A.L., Attwood T.K., Babbitt P.C., Blum M., Bork P., Bridge A., Brown S.D., Chang H.Y., El-Gebali S., Fraser M.I. (2019). Interpro in 2019: Improving coverage, classification and access to protein sequence annotations. Nucleic Acids Res..

[31] Richardson L.J., Rawlings N.D., Salazar G.A., Almeida A., Haft D.R., Ducq G., Sutton G.G., Finn R.D. (2019). Genome properties in 2019: A new companion database to interpro for the inference of complete functional attributes. Nucleic Acids Res..

[32] Jayanna P.K., Bedi D., Deinnocentes P., Bird R.C., Petrenko V.A. (2010). Landscape phage ligands for pc3 prostate carcinoma cells. Protein Eng. Des. Sel..

[33] Fagbohun O.A., Bedi D., Grabchenko N.I., Deinnocentes P.A., Bird R.C., Petrenko V.A. (2012). Landscape phages and their fusion proteins targeted to breast cancer cells. Protein Eng. Des. Sel..

[34] Petrenko V.A., Smith G.P. (2000). Phages from landscape libraries as substitute antibodies. Protein Eng..

[35] Fagbohun O.A., Kazmierczak R.A., Petrenko V.A., Eisenstark A. (2013). Metastatic prostate cancer cell-specific phage-like particles as a targeted gene-delivery system. J. Nanobiotechnol..

[36] Smith G.P., Petrenko V.A., Matthews L.J. (1998). Cross-linked filamentous phage as an affinity matrix. J. Immunol. Methods.

[37] Cailleau R., Young R., Olive M., Reeves W.J. (1974). Breast tumor cell lines from pleural effusions. J. Natl. Cancer Inst..

[38] Gillespie J.W., Wei L., Petrenko V.A. (2016). Selection of lung cancer-specific landscape phage for targeted drug delivery. Comb. Chem. High Throughput Screen..

[39] Teyra J., Huang H., Jain S., Guan X., Dong A., Liu Y., Tempel W., Min J., Tong Y., Kim P.M. (2017). Comprehensive analysis of the human sh3 domain family reveals a wide variety of non-canonical specificities. Structure.

[40] Saksela K., Permi P. (2012). Sh3 domain ligand binding: What’s the consensus and where’s the specificity?. FEBS Lett..

[41] Kurochkina N., Guha U. (2013). Sh3 domains: Modules of protein-protein interactions. Biophys. Rev..

[42] Stoll R., Bosserhoff A. (2008). Extracellular sh3 domain containing proteins—Features of a new protein family. Curr. Protein Pept. Sci..

[43] Pornillos O., Alam S.L., Davis D.R., Sundquist W.I. (2002). Structure of the tsg101 uev domain in complex with the ptap motif of the hiv-1 p6 protein. Nat. Struct. Biol..

[44] Sundquist W.I., Schubert H.L., Kelly B.N., Hill G.C., Holton J.M., Hill C.P. (2004). Ubiquitin recognition by the human tsg101 protein. Mol. Cell.

[45] Ahmed I., Akram Z., Iqbal H.M.N., Munn A.L. (2019). The regulation of endosomal sorting complex required for transport and accessory proteins in multivesicular body sorting and enveloped viral budding—An overview. Int. J. Biol. Macromol..

[46] Dussupt V., Javid M.P., Abou-Jaoude G., Jadwin J.A., de La Cruz J., Nagashima K., Bouamr F. (2009). The nucleocapsid region of hiv-1 gag cooperates with the ptap and lypxnl late domains to recruit the cellular machinery necessary for viral budding. PLoS Pathog..

[47] Irie T., Harty R.N. (2005). L-domain flanking sequences are important for host interactions and efficient budding of vesicular stomatitis virus recombinants. J. Virol..

[48] Licata J.M., Simpson-Holley M., Wright N.T., Han Z., Paragas J., Harty R.N. (2003). Overlapping motifs (ptap and ppey) within the ebola virus vp40 protein function independently as late budding domains: Involvement of host proteins tsg101 and vps-4. J. Virol..

[49] Ivanenkov V.V., Menon A.G. (2000). Peptide-mediated transcytosis of phage display vectors in mdck cells. Biochem. Biophys. Res. Commun..

[50] Park J.E., Soung N.K., Johmura Y., Kang Y.H., Liao C., Lee K.H., Park C.H., Nicklaus M.C., Lee K.S. (2010). Polo-box domain: A versatile mediator of polo-like kinase function. Cell Mol. Life Sci..

[51] Lee S.Y., Jang C., Lee K.A. (2014). Polo-like kinases (plks), a key regulator of cell cycle and new potential target for cancer therapy. Dev. Reprod..

[52] Kumar S., Sharma A.R., Sharma G., Chakraborty C., Kim J. (2016). Plk-1: Angel or devil for cell cycle progression. Biochim. Biophys. Acta Rev. Cancer.

[53] Durocher D., Jackson S.P. (2002). The fha domain. FEBS Lett..

[54] Perou C.M. (2010). Molecular stratification of triple-negative breast cancers. Oncologist.

[55] Ozbabacan S.E.A., Engin H.B., Gursoy A., Keskin O. (2011). Transient protein-protein interactions. Protein Eng. Des. Sel..

[56] Yu J.N., Smith G.P. (1996). Affinity maturation of phage-displayed peptide ligands. Method Enzymol..

[57] Petrenko V.A., Gillespie J.W. (2017). Paradigm shift in bacteriophage-mediated delivery of anticancer drugs: From targeted magic bullets to self-navigated magic missiles. Expert Opin. Drug Deliv..

[58] Kim E.S. (2008). Directed evolution: A historical exploration into an evolutionary experimental system of nanobiotechnology, 1965–2006. Minerva.

[59] Marvin D.A., Hale R.D., Nave C., Helmer-Citterich M. (1994). Molecular models and structural comparisons of native and mutant class i filamentous bacteriophages ff (fd, f1, m13), if1 and ike. J. Mol. Biol..

[60] Marvin D.A., Symmons M.F., Straus S.K. (2014). Structure and assembly of filamentous bacteriophages. Prog. Biophys. Mol. Biol..

