# Supplementary material for: Combinatorial Avidity Selection of Mosaic Landscape Phages Targeted at Breast Cancer Cells—An Alternative Mechanism of Directed Molecular Evolution
Source: Viruses. 2019 Aug 26;11(9):785. doi: 10.3390/v11090785 (PMC6784196; doi:10.3390/v11090785)
Supplement: Supplementary file 1 [file viruses-11-00785-s001.pdf]

Table S1: Target domains identified from selected phages

Partner Domain		Function [Location]	CorM	SliM[a]	Phage
**A**	**Atg8**[b]	LC3-interacting region (LIR) mediates binding to Atg8 ubiquitylated proteins [membrane]	STL,AEY<br>VND<br>ESW,SWD<br>DYD<br>EYG,GES,VNA,SVN	EYSTL<br>EYNMV<br>DFSTP<br>DYDMI<br>GESVNA	ADHAEYSTL<br>DFEYNMVND<br>DFSTPESWD<br>VDYDMIGDQ<br>AEYGESVNA
**B**	**PDZ**	Autophagy-related [membrane-associated complexes]	EYG,GES,VNA,SVN<br>SVD	VNA<br>QSSVDA	AEYGESVNA<br>GDYQSSVDA
**C**	**WD40** domain of **WDR5**	Mediate assembly of histone modification complexes [nucleus]	VDV<br>SVD<br>PPT,APE	DYVDV<br>QSSVDA<br>PTAP	DYVDVSIND<br>GDYQSSVDA<br>DFPPTAPED
**D**	**SUMO** membrane protein	Sorting and internalization signal	LLN,LNE<br>LLN,LNE	DTIALL<br>ELEHLLN	DDTIALLNE<br>EELEHLLNE
**E**	nuclear receptors	Expression of specific genes, development, homeostasis, and metabolism	LLN,LNE	ELEHLLN	EELEHLLNE
**F**	**UEV**	Nucleus, ESCRT-I complex[c] [cytosol]	DFP,FPP,PPT,APE	PPTAPE	DFPPTAPED
**G**	**SH3**	Signal transduction, traffic, cytoskeleton and organelle organization [cytosol]	DFP,FPP,PPT,APE<br>DSF,FVN,VNA	DFPPTAP<br>DSFVNAP	DFPPTAPED<br>DSFVNAPED
**H**	**mu subunit** Adaptor Protein **AP**	Proteins directing traffic within the endosomal and the secretory pathways	DYD<br>VND	DYDMI<br>YNMV	VDYDMIGDQ<br>DFEYNMVND
**I**	**MYND** zinc	gene regulation, cancers	LEP,EPG,GQD	PPLEP	GPPLEPGQ
**J**	**MATH**[d]	deubiquitinating protease **USP7**[c]**;** substrate recognition, nuclear localization	DSY<br>DDS,DSY	PLDSY<br>PDDSY	GTGPLDSYD<br>VHPDDSYSD
**K**	**BIR**[f] motif in **IAP**[e] Proteins	involved in regulation of apoptosis	DAD,DPS	DADPS	VPSYDADPS

[a] Short Linear Motifs (SLiMs) discovered using the Eukaryotic Linear Motif (ELM) resource (Gouw, et al., 2018)

[b] Atg8 - Autophagy-related protein

[c] USP7 - ubiquitin specific protease 7

[d] MATH - Meprin And TRAF-Homology (MATH) domain

[e] IAP - Inhibitor of Apoptosis Proteins

[f] BIR - Baculovirus Inhibitor of apoptosis protein Repeat
